# Salubrious effects of *Ficus carica* L. leaves extract in inflammation, diabetes, and obesity: An in-vitro, in-silico, and in-vivo study

**DOI:** 10.29219/fnr.v70.11024

**Published:** 2026-02-19

**Authors:** Syed Zia ul Hasnain, Maryam Ahmed, Adeola Tawakalitu Kola-Mustapha, Jahanzeb Mudassir, Ambreen Aleem, Iqra Islam, Adnan Amin, Khizar Abbas, Asad Saleem Sial

**Affiliations:** 1 Department of Pharmacognosy, Faculty of Pharmacy and Pharmaceutical Sciences, University of Karachi, Karachi, Pakistan; 2 College of Pharmacy, Alfaisal University, Riyadh, Saudi Arabia; 3 Department of Pharmaceutics and Industrial Pharmacy, University of Ilorin, Nigeria; 4 Facultyof Pharmacy, Bahauddin Zakariya University, Multan, Pakistan; 5 Department of Pharmacognosy, Gomal University, Dera Ismail Kan, Pakistan

**Keywords:** antidiabetic activity, antioxidant activity, anti-inflammatory activity, phytochemical constituents, hypolipidemic

## Abstract

Various traditional medicinal systems have utilized the plant-based remedies for addressing the diverse ailments worldwide. Hence, this study aimed to scientifically explore the biological and phytochemical potential of *Ficus carica* L. leaves. This investigation encompassed the assessments of flavonoids, total phenolic contents, as well as physicochemical and phytochemical properties. Antioxidant potential was evaluated through hydrogen peroxide, 2,2-diphenyl-1-picrylhydrazyl (DPPH), and ferric reducing antioxidant power (FRAP) assays, while anti-inflammatory effects were determined *via* proteinase inhibition, bovine serum albumin (BSA) denaturation, and heat-induced hemolysis assays. Additionally, antiglycation potential was assessed through free carbonyl group estimation, fructosamine, and Congo-red assays. The impact on diabetes mellitus, obesity, and renal and hepatic functions was investigated using the high-fat high-sugar diet model. Advanced analytical techniques including Fourier-transform infrared, high-performance liquid chromatography, and liquid chromatography-tandem mass spectrometry were employed to identify the active secondary metabolites present in the *F. carica* L. leaf extract. Molecular docking and absorption–distribution–metabolism–excretion–toxicity analyses were performed by different computational methods. Results revealed that substantial levels of total flavonoids (123 mg rutin equivalents/g) and phenolic content (333 mg gallic acid equivalent/g) along with promising antioxidant activity (IC_50_: 0.58 mg/mL for DPPH assay, 35.6% inhibition for H_2_O_2_ assay, and FRAP value of 88.769 µg/g Fe_2_SO_4_ solution) were found. Notably, *F. carica* L. leaf extract exhibited the significant inhibition in heat-induced hemolysis (55 ± 0.03%), proteinase activity (28 ± 0.01%), and BSA denaturation (51.2 ± 0.05%). Furthermore, it exhibited the significant therapeutic effects on the biomarkers related to diabetes mellitus, obesity, liver, and kidney functions. Chemical analyses unveiled the presence of chlorogenic acid, ferulic acid, thymoquinone, rutin, coumarin, as well as terpenoids, alkaloids, coumarins, and flavonoids. The key findings suggest that *F. carica* L. leaf extract holds significant potential as an antioxidant, antidiabetic, and hypolipidemic agent.

## Popular scientific summary

- Our study explores the therapeutic potential of *Ficus carica* L. (fig) leaves in managing diabetes, obesity, and inflammation. Obesity and diabetes are major global health concerns, often leading to serious complications like heart disease and liver dysfunction. We analyzed fig leaf extract using advanced techniques (HPLC, LC-MS/MS, FTIR) and found it rich in flavonoids and phenolic compounds with strong antioxidant, anti-inflammatory and antidiabetic properties. In diabetic and obese rats, the extract significantly lowered blood sugar, reduced inflammation, improved liver and kidney function, and aided weight management. Molecular docking studies further confirmed its potential in targeting key metabolic pathways. Our findings suggest that fig leaf extract could serve as a natural alternative for managing metabolic disorders. With further research, it may contribute to developing plant-based therapies for diabetes, obesity, and inflammation.

Obesity is a complex chronic disease associated with significant mortality and morbidity due to its direct connection with various metabolic and inflammatory disorders. This condition is characterized by an inflammatory state, marked by a simultaneous decrease in anti-inflammatory cytokines such as adiponectin and interleukin (IL)-10, along with an increase in the secretion of pro-inflammatory molecules, including leptin, IL-8, IL-6, tumor necrosis factor-*α*, and IL-1*β* (1). The pathogenesis of obesity involves multiple factors, including impaired neuroendocrine feedback, altered brain circuits, and an imbalance between energy expenditure and intake (2). These complications encompass type 2 diabetes mellitus (T_2_DM), non-alcoholic fatty liver disease, dyslipidemia, autoimmune conditions, altered immune responses, sleep apnea, cancer, renal disorders, and cardiovascular diseases (3, 4).

The prevalence of obesity has surged globally, emerging as a significant public health concern exacerbated by the industrialization of food production and increasingly sedentary lifestyles (3). T_2_DM stands out as a primary metabolic complication of obesity, characterized by inadequate insulin secretion and insulin resistance in peripheral areas such as the liver, adipose tissues, and skeletal muscle (2). The quest for innovative and alternative pharmaceuticals with antiadipogenic, lipolytic, and antidiabetic properties derived from natural sources has gained significant attraction. This approach aims to mitigate the drawbacks associated with synthetic antidiabetic medications, including drug resistance, side effects, and toxicity (5). A significant proportion of the human diet comprises vegetables and fruits, recognized as vital sources of phytochemicals. These compounds exhibit a wide array of properties, including antiviral, anti-inflammatory, antioxidant, antithrombotic, antibacterial, cholesterol-lowering, and antifungal effects (6). Phytochemicals exert their effects on the human body through mechanisms analogous to those of known chemical compounds.

*Ficus carica* L., belongs to the Moraceae family, is one of the oldest cultivated seasonal fruit-bearing plants, originating from the Middle East and comprising over 800 species of epiphytes, shrubs, and trees. Traditionally, it has been utilized for treating various ailments, such as ulcers, cancer, DM, inflammation, menstrual pain, paralysis, scabies, asthma, and gonorrhea. The plant exhibits antipyretic, purgative, aphrodisiac, antioxidant, antimicrobial, antifungal, and hypotensive properties and is used for managing cough and vomiting (7). Chemically, *Ficus carica* contains a plethora of compounds, including ceramides, cerebrosides, steroids, pentacyclic triterpenes, flavonoids, fibers, chlorogenic acid, rutin, luteolin, (+)-catechin (8), psoralen, psoralic acid-glucoside, caffeoylmalic acid, minerals, vitamins, *β*-amyrins, arabinose, *β*-carotene, *β*-sitosterols, glycosides, carbohydrates, polyphenols, organic acids, sugars (9), phenolic compounds, phytosterols, triterpenoids, coumarins, and aliphatic alcohols (6).

Despite its extensive use, there is a lack of literature information for the assessment of estimation of secondary metabolites, including phenolic and flavonoid compounds, along with their antioxidant potential, biological activities, as well as the utilization of advanced analytical techniques, including Fourier-transform infrared (FTIR), high-performance liquid chromatography (HPLC), and liquid chromatography–tandem mass spectrometry (LC-MS/MS) techniques on the extract of *F. carica* leaves. Consequently, this study was planned with the objective to conduct the phytochemical analysis using advanced analytical techniques, including FTIR, HPLC, and LC-MS/MS techniques. Furthermore, we also evaluated of antioxidant, hypoglycemic, hypolipidemic, and anti-inflammatory properties.

## Materials and methods

All the chemicals used in this study were of analytical grade. Creatinine, aspartate aminotransferase (AST), total bilirubin, alanine transferase, blood urea nitrogen (BUN), and alkaline phosphatase (ALP) assay kits were purchased from HUMAN diagnostics, Germany. The triglycerides (TG) assay kit was purchased from Clinical-Systems, India. Total cholesterol (TC) and high-density lipoprotein (HDL) kits were purchased from Bio-Systems S.A, Barcelona, Spain.

### Collection of plant

*Ficus carica* L. leaves were collected from a locality of Sahiwal, Pakistan, and were subjected to identification by Prof. Dr. Zafarullah Zafar of Department of Botany, Bahauddin Zakariya University, Multan, Pakistan, and the sample was retained in herbarium of Department of Pharmacognosy, Faculty of Pharmacy, Bahauddin Zakariya University, Multan, Pakistan, under the specimen voucher number (www.theplantlist.org/tpl1.1/record/kew-2809827). Around 5 Kg of the leaves of *F. carica* L. deprived of stalks were shade dried, weighed, and coarsely grinded to form the powder.

### Physicochemical analysis

#### Loss on drying

Approximately 2.0 gm of the leaf powder of *F. carica* L. was placed into a porcelain dish. Oven was used to dry the powdered drug at 105°C to obtain the constant weight. The desiccator was used to cool the powdered drug. The weight loss was usually considered as the presence of moisture and was find out by applying the following equation as described previously with some modifications:


$$\text{Loss on drying}=\frac{\text{Weight of powder after heating}}{\text{Initial weight of leaf powder}}\times 100$$ (Eq. 1)

#### Total ash

A quantity of 2.5 g of leaf powder was placed in a crucible, which was then subjected to a temperature range of 550–600°C for the duration of 2–3 h within a muffler furnace. Subsequently, the furnace was allowed to cool, and the resulting ash was weighed. This process was repeated continuously until a constant weight was achieved. The total ash content was quantified using the following equation as described previously with some modifications:


$$\%\text{age Total ash}=\frac{\text{Weight of total ash}}{\text{weight of leaf powder}}\times 100$$ (Eq. 2)

#### Acid-insoluble ash

Approximately 25 mL of 10% HCl was mixed with the total ash present in an ash crucible, heated for 5 min at 500–600°C, and then filtered the mixture through filter paper. After drying and washing the residues with hot distilled water, the percentage acid-insoluble ash was calculated by applying the following equation:


$$\%\text{age Acid-isoluble ash}=\frac{\text{Weight of acid}-\text{insoluble ash}}{\text{Initial weight of leaf powder}}\times 100$$ (Eq. 3)

## Water-soluble ash

Total ash of the powdered drug was placed in a beaker containing 20 mL water. The beaker was placed in water bath to boil the mixture. Then, after boiling, the filter paper was used to filter the mixture, and residue was washed two times with hot water. Filter paper was placed on an ash crucible and heated the crucible at the temperature of 500–600°C for 5 min. Thereafter, desiccator was used to cool the product. The ash content was calculated using the following equation:


$$\%\text{age Water-soluble ash}=\frac{\text{Weight of total ash}-(\text{Weight of water}-\text{insoluble ash})}{\text{Initial weight of leaf powder}}\times 100$$ (Eq. 4)

### Preparation of extract

Approximately 1,000 g of coarsely ground powder of *F. carica L*. leaves was macerated in 1.5 L of 98% ethanol for the duration of 7 days. Afterward, the soaked material underwent filtration using the Whatman filter paper, resulting in the separation of solid residue and filtrate. The solid residue was subjected to two additional rounds of maceration, first with 1.0 L and then with 0.8 L of 98% ethanol to complete the extraction process. Subsequently, the filtrate was obtained. Then, all the three filtrates were combined and subjected to drying with rotary evaporator (Buchi Switzerland) at a pressure of 74.51 torr, a rotation speed of 4 RPM, and temperature conditions between 30 and 40°C. A thick, viscous paste was weighed to ascertain the percentage yield, placed in a closed container, labeled, and preserved at −20°C in Biomedical Freezer (Haier Biomedical, DW-25L262, Japan) for further analysis.

### Phytochemical profiling

Phytochemical profiling of *F. carica* L. leaves ethanolic extract (FLE) was performed to detect and confirm the occurrence of primary and secondary metabolites following the methods as described previously (10) with some modifications.

### Total phenolic contents

The Folin-Ciocalteu’s reagent method was used to estimate the total phenolic contents. Briefly, the FLE solution was prepared in 0.2–1 mg/mL, and around 1 mL sample of FLE was mixed with 1.0 mL Folin-Ciocalteu’s reagent. After 5 min of incubation, 10 mL of 7% Na_2_CO_3_ solution and 13 mL of distilled water were thoroughly mixed and added to the mixture. The incubation of reaction mixture was performed in a dark room for an hour, and the absorbance was recorded at 750 nm using UV/Vis. spectrophotometer (Optima, SP-3000, Tokyo, Japan). Gallic acid was used as a standard, and the results were expressed as (mg GAE/g) of extract and were calculated using the following equation by following the method described previously with some modifications:


$$\text{Total phenolic content}=\frac{\text{C}\times \text{DF}\times \text{V}}{\text{m}}$$ (Eq. 5)

where C is the concentration of gallic acid, DF is the dilution factor, V is the volume of extract, and m is the weight of sample.

### Total flavonoids contents

The protocol as described by Aslam et al. with slight modifications was used to determine the presence of total flavonoids contents in FLE (11). Rutin was utilized as a reference drug to calculate the total flavonoid contents. Approximately 1 mL of crude extract was mixed in 0.15 mL of 0.5 M NaNO_2_ and 0.15 mL of 0.3M AlCl_3_.6H_2_O. Then, the mixture was uniformly mixed with 1 mL of 1M NaOH. UV/Vis. spectrophotometer was used to measure the absorbance at 506 nm after 5 min, and results were calculated using the following equation and were presented as (mg RE/g) of extract:


$$\text{Total flavonoids content}=\frac{\text{C}\times \text{DF}\times \text{V}}{\text{m}}$$ (Eq. 6)

where C is the concentration of rutin calculated from standard curve, DF is the dilution factor, V is the volume extract, and m is the weight of sample.

### Estimation of hydrogen peroxide scavenging activity

The technique described by Akhter et al. in 2021 was slightly modified to assess the H_2_O_2_ scavenging potential of FLE (12). The H_2_O_2_ scavenging activity of FLE was evaluated at doses ranging from 0.5 to 8 mg/mL. A sample solution (1 mL) was incubated with 0.6 mL of 40 mM H_2_O_2_. After 10 min, a UV/Vis. spectrometer was used to measure the absorbance of hydrogen peroxide at 230 nm. Gallic acid was used as the standard. The hydrogen peroxide scavenging potential of FLE was determined using the following equation:


$${\text{H}}_{2}{\text{O}}_{2}\text{ Inhibition capacity}=1-\frac{\text{Absorbance of Sample}}{\text{Absorbance of Blank}}\times 100$$ (Eq. 7)

### Estimation of free radical scavenging activity by DPPH

FLE scavenging activity by the 2,2-diphenyl-1-picrylhydrazyl (DPPH) method was calculated by applying the Wintola et al. method with slight amendments (13). 0.2–1 mg/mL of gallic acid and FLE were prepared by using methanol as a solvent. 1 mL of each dilution and 1.0 mL of 0.135 mM DPPH solution prepared in methanol were mixed by using vortex mixture and incubated in a dark room for 30 min at 25°C. The absorption of each sample was determined spectrophotometrically at 517 nm wavelength with the help of UV/Vis. spectrometer. The %inhibition was calculated using the following equation:


$$\%\text{ Inhibition}=\frac{\text{Absorbance of Control}-\text{Absorbance of Sample}}{\text{Absorbance of Control}}\times 100$$ (Eq. 8)

### Estimation of antioxidant activity by Ferric reducing antioxidant power assay

The Ferric Reducing Antioxidant Power (FRAP) assay was used by following the protocol as described previously (14) with some modifications. 2.5 mL of 20 mM/L of the FRAP reagent solution, 25 mL of 0.3 mol/L acetate buffer, and 2.5 mL of 10 mM/L of TPTZ (2,4,6-tri-2-pyridinyl-1,3,5-triazine) were prepared at a temperature of 37°C. 40 μL of sample solution was diluted with 0.2 mL distilled water and then mixed with 1.8 mL of the FRAP reagent. The reaction mixture was incubated for 10 min at 37°C, and the absorbance was measured using a UV/Vis. spectrophotometer at 593 nm. FeSO_4_ (1 mM/L) served as the reference standard. A standard curve of FeSO_4_ was plotted, and the results of the assay were presented as the extract concentration having ferric-reducing ability equivalent to that of 1 mM/L FeSO_4_.

### Estimation of proteinase inhibition activity

The proteinase inhibition assay was conducted following a modified protocol as described previously (15). Initially, 0.06 mg trypsin, 1 mL Tris-HCl buffer (20 mM, pH 7.4), and FLE extract solution (1.0 mL) at various concentrations (100–500 µg/mL) were combined to form a 2.0 mL mixture, which was then incubated at 37°C for 5 min. Subsequently, 1.0 mL of 0.8% (w/v) casein was added to the reaction mixture and allowed to incubate for 20 min. Following this, 2 mL of 70% perchloric acid was added, resulting in the formation of a cloudy solution. The solution was then centrifuged, and the absorbance of the supernatant was measured at 210 nm using a UV/Vis. spectrophotometer. The %inhibition was calculated using the following equation:


$$\%\text{ Inhibition}=\frac{\text{Absorbance of Control}-\text{Absorbance of Sample}}{\text{Absorbance of Control}}\times 100$$ (Eq. 9)

### Analysis of heat-induced hemolysis

The heat-induced hemolysis assay was conducted using a modified method as described previously (16). Initially, a suspension of blood cells (0.05 mL) and FLE solution (0.05 mL) prepared in distilled water were combined with 2.95 mL of phosphate buffer (pH 7.4). The resulting reaction mixture was then subjected to a shaking water bath for 20 min at 54°C. Subsequently, the mixture was centrifuged for 3 min at 2,500 rpm, and the absorbance of the supernatant at 540 nm was measured using a UV/Vis. spectrophotometer. A solution of phosphate buffer served as the experimental control. The hemolysis level was calculated using the following equation:


$$\%\text{ inhibition of hemolysis}=100\times \frac{1-\text{Absorbance of sample}}{\text{Absorbance of Control}}$$ (Eq. 10)

### Analysis of protein denaturation

The assay was conducted following the method as described previously (17) with some modifications. Briefly, a mixture of 1% bovine albumin (0.2 mL), phosphate-buffered saline (4.780 mL, pH 6.4), and FLE at a concentration of 5 mg/mL (0.02 mL) was gently mixed and incubated for 15 min in a water bath at 37°C. Subsequently, the reaction mixture was heated at 70°C for 5 min. Following the cooling of the reaction mixture, the absorbance at 660 nm was measured using a UV/Vis. spectrophotometer. The %inhibition of protein denaturation was calculated using the following equation:


$$\%\text{ inhibition of denaturation}=100\times \frac{1-\text{Absorbance of sample}}{\text{Absorbance of Control}}$$ (Eq. 11)

### Detection of *β*-amyloid formation

This assay was used to measure the aggregation in FLE-glycated sample, according to the protocol as described previously (18). Briefly, phosphate buffer solution (400 µl) of pH 7.4 and Congo-red solution (100 µL) were mixed with 100 µL of FLE (0.25 mg/mL) and/or control (bovine serum albumin (BSA)+ Glucose). The reaction mixture was incubated for 20 min in a dark rooms, and the absorbance at 530 nm was measured using a UV/Vis. spectrophotometer.

### Fructosamine assay

The concentration of both the control and fructosamine (amadori product in glycated albumin samples) was determined using the method as described previously (19) with some modifications. A solution of nitro blue tetrazolium (NBT) (0.75 mM) was prepared in 0.1 M carbonate buffer (pH 10.35). 0.8 mL of NBT solution was incubated with glycated samples (40 µL) for 30 min at 37°C. The absorbance at 530 nm was measured using a UV/Vis. spectrophotometer. The fructosamine concentration was determined with the help of typical 1-deoxy-1-moepholinofructose curve and expressed in µM/mg of protein (*r* = 0.981, Y = 0.00X + 0.017), and results are displayed in Table 5.

### Free Carbonyl Group estimation

The Ashraf et al.’s method was used to access the carbonyl group in glycated test samples (20). HCl 2.5 M was used to prepare 10 mM DNPH (2,4-Dinitrophenylhydrazine). The procedure involved mixing 500 µL of the glycated sample with 500 µL of DNPH solution, followed by incubation for 60 min at 25°C. Subsequently, precipitation was initiated by adding 1.0 mL of 20% trichloroacetic acid. Washing of precipitate was carried out with 1 mL mixture composed of ethanol:ethyl acetate in 1:1 (v/v), and 1 mL 6M urea was used to dissolve pellets. After that, UV/Vis. spectrometer was used to measure absorbance at 365 nm. The molar extinction coefficient (*ε*) at 365 nm of 21 mM^-1^ cm^-1^ was employed to compute the concentration of protein carbonyl groups, expressed as nM/mg of protein.

### In-vivo methodology

Wistar albino rats of both sexes, with a body weight ranging from 120 ± 20 g, were accommodated in the animal facility at the Faculty of Pharmacy, Bahauddin Zakariya University, Multan, Pakistan. All rats were housed in polycarbonate cages measuring 18 × 34 × 7 cm^3^, with a maximum of six rats per cage, under standard conditions of temperature (25 ± 2°C) and humidity, with a 12–12-h light-dark cycle. They were provided with conventional animal food and *ad libitum* access to water. The experimental protocol was approved by the Institutional Animal Ethics Committee at the Faculty of Pharmacy and Pharmaceutical Sciences, University of Karachi. The experiment was conducted in compliance with the committee’s regulations for the supervision and management of animal experiments, as per Institutional Bioethical Committee Approval No. IBC KU-289/2022.

Animal study was performed according to the standard protocol with slight modifications. Wistar albino rats (*n* = 48) weighing (120 ± 20 g) were acclimatized for 15 days before the start of the experiment, and then the following treatments were given to rats of each group.

Group A: It was called as normal control (NC), consisted of six animals, and received normal saline at the rate of 5 mL/kg of body weight per-oral for complete period of study.

Group B: It consisted of 42 rats, received 3 mL/kg body weight of high-fat high-sugar diet (HFHSD) that composed of Vanaspati ghee and coconut oil (3:1) and 10 mL/kg 25% dextrose water P.O. for 8 weeks, and then divided into following different groups.

Group B1: It was called as the obesity control group and consisted of six animals. All animals were fed with 3 mL/kg of body weight HFHSD for remaining 4 weeks of study.

Group B2: It was called as the obesity treatment (OT) group; rats were fed with HFHSD continuously along with atorvastatin (standard drug) 60 mg/kg of body weight orally for a period of 4 weeks.

Group B3: It was called as the obesity diabetes group; rats (*n* = 30) were fed with HFHSD along with streptozotocin (65 mg/kg) of body weight interperitoneally prepared in citrate buffer of pH 4.5. The blood glucose level was checked after 48 h after injection. Animals showing blood glucose level ≥200 mg/mL were randomly divided into following five groups (*n* = 6) and were fed with HFHSD for a complete period of study (12 weeks).

Group C: It was called as the obesity diabetes control (ODC) group and was administered normal saline orally 5 mL/kg of body weight for a period of 4 weeks.

Group D: It was called as the obesity diabetes treatment (ODT) group; rats in this group were administered with glibenclamide (10 mg/kg of body weight) and atorvastatin (60 mg/kg of body weight for 4 weeks).

Group E: It was called as the obesity diabetes treatment (ODFLE1) group, in which rats were fed with FLE (100 mg/kg of body weight) orally for 4 weeks.

Group F: It was called as the obesity diabetes treatment (ODFLE2) group; in this group, rats were treated with FLE (250 mg/kg body weight) for a period of 4 weeks.

Group G: It was called as the obesity diabetes treatment (ODFLE3) group, in which rats were administered with FLE (500 mg/kg) of body weight for 4 weeks (21). Body weight and blood sugar level were measured at the 1st day, 8 weeks, 8 week and 2 days, and 12 weeks.

### Biochemical analysis

Blood samples for liver function test (aspartate transaminase, ALP, and alanine transaminase), lipid profile (low-density lipoproteins (LDL), HDLs, TG, and TC), and Renal Function tests (RFTs) (creatinine, urea, and uric acid) were collected by closed cardiac puncture at start, after 8 weeks of HFHSD, and at the end of study period and preserved at −20°C. The body weight (bwt) of all animals was also measured at the start, after 8 weeks of HFHSD, after 8 weeks and 2 days, and then at the end of study period.

### Statistical analysis

The results obtained from the research were presented as mean ± standard error of the mean (SEM) of *n* = 6 in each group. GraphPad Prism 9.0.1 (La Jolla, CA, USA) was utilized to create the graphs, and statistical analysis was performed using two-way ANOVA followed by Tukey’s test to determine the significance between different groups, with a significance level set at *P* ≤ 0.05.

### FTIR analysis

The FTIR spectroscopy was used to identify various functional groups present in FLE. 0.5 g of FLE was placed on the Attenuated Total Reflectance (ATR)-FTIR of the Bruker IR Affinity 1 Model, Japan, limited with a wavelength of 400–4,000 cm-1 equipped with Bruker OPUS software. IR light was absorbed by the sample at different wavelengths, and this absorbed energy converted into the vibrational energy, which forms the signals; then, a detector is used to analyze. The peaks obtained were interpreted on screen of computer, and different functional groups were analyzed (22).

### HPLC analysis

HPLC analysis was performed on a Spectra-Physics SP 8800/8810LC pump coupled to a Varian 9065 polychrom diode-array detection system. The mobile phase consisted of acetonitrile and distilled water in a ratio of 60:40 (ACN:H2O), with a flow rate of 1 mL/min. A volume of 500 µL of FLE solution (5 mg/mL) was injected onto a Supelco® HPLC column (12 × 260 mm, 5 µm, Sigma Aldrich), and separation was achieved using a 17-min linear gradient at 25°C (23).

For analysis, the following phenolic compounds were employed: chlorogenic acid, ferulic acid, rutin, coumarin, and thymoquinone. The peaks present in the samples were compared with those of the standards for identification.

### LC-CMS/MS analysis

LC-MS/MS analysis was performed using an ion trap tandem mass spectrometer (AmaZon speed, Bruker Daltonics, Bremen, Germany) hyphenated with a ThermoFischer Scientific UHPLC system (Bremen, Germany). 10 μL extract (1 mg/mL) was injected into the Agilent Technologies Zorbax SB-C18 column (50 mm × 2.1 mm, 1.8 m, CA, USA) via autosampler for a gradient separation. Separation was achieved using a flow rate of 0.2 mL/min of eluent A (0.1% formic acid in water) and eluent B (0.1% formic acid in methanol), changing from 5% B to 100% B in 20 min. 100% B was maintained for 5 min before returning to 5% B. Compounds were ionized using Electrospray ionization (ESI) ion source operation in positive ion mode at 4,500 V. Other ion source parameters were as follows: dry gas pressure was 10 psi, dry gas flow was 4 L/min, and dry gas temperature was 180°C. The mass scan range was 100 to 2,000 amu. The top 10 precursor ions based on intensities were selected and fragmented in an automatic fashion. The MS/MS scan range was 50–2,000 amu. The MS/MS data underwent processing by utilizing Compass Data Analysis 4.4 (Bruker Daltonics). All MS/MS spectra were converted to the mgf format. Mgf files were then searched in Global Natural Product Social Molecular Networking Libraries Search using default search parameters, that is, MS tolerance of 02 amu and MS/MS tolerance of 0.5 amu with at least 3 peaks matching, and a score threshold of 0.8 MS/MS matching was then manually validated (24).

### Molecular docking and absorption–distribution–metabolism–excretion–toxicity profiling

Molecular docking may be employed to validate vital targets in network pharmacology. We first glanced the PubChem database for key molecules and then exported the structure of compounds. Next, the core proteins’ 3D structures were retrieved from the Protein Data Bank (https://www.rcsb.org/). The progression of dehydration, hydrogenation, and charge editing was then tracked with AutoDock 4.2.6, and the changed target proteins and key compounds were saved as PDBQT files. Ultimately, the docking outcomes were loaded into the PyMol software for assessment and visualization (25).

The Swiss ADME internet server was used to do computer learning to predict drug-like characteristics. Five important substances were entered into the Swiss ADME website, and absorption–distribution–metabolism–excretion–toxicity (ADMET) properties were chosen for analysis. The toxicity of the five most relevant substances was predicted using the open-source website ProTox-II, and the results were reported.

## Results

**Fig. 1:**
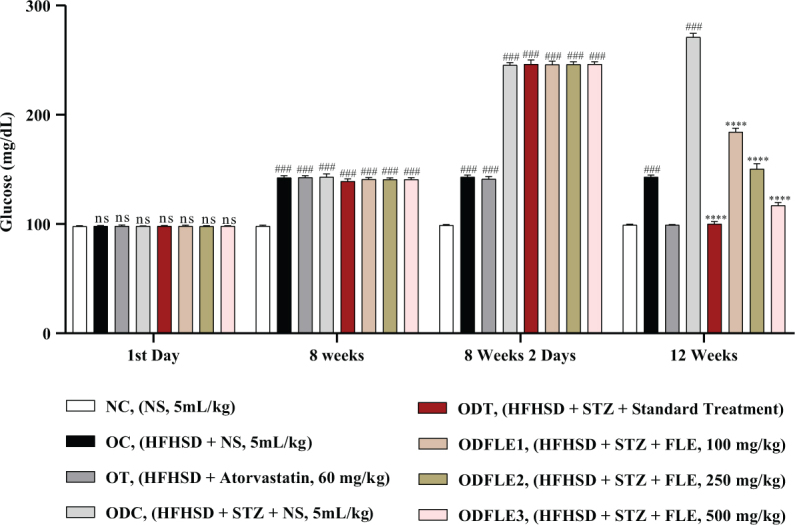
Changes in glucose levels of *Wistar albino* rats STZ was administered via the intraperitoneal route after obesity induction in rats by feeding with HFHSD for 8 weeks followed by atorvastatin 60 mg/Kg bwt, glibenclamide 10 mg/Kg bwt and FLE doses at 100 mg/Kg bwt, 250 mg/Kg bwt and 500 mg/Kg bwt along with HFHSD for 4 weeks’ treatment period. (a) Glucose was measured with glucometer. Mean ± SEM of *n* = 6. Each group is analyzed using two-way ANOVA followed by Tukey’s test. At 1st day all the groups showed non-significant (ns) variations as compared to normal control (NC) group. After 8 weeks’ period, all the groups showed very significant (###) with *P* < 0.001 as compared to NC group. After 8 weeks’ 2 days period, all the groups showed very significant (###) with *P* < 0.001 as compared to NC group. After 12 weeks’ period, when the obesity control (OC) group was compared with obesity treatment (OT) group and obesity diabetes control (ODC) group is compared to treatment groups (ODT, ODFLE1, ODFLE2 and ODFLE3), results are considered non-significant (ns) if *P* > 0.05, significant (*) if *P* < 0.05, more significant (**) if *P* < 0.01, very significant (***) if *P* < 0.001, and highly significant (****) if *P* < 0.0001. When the NC group is compared with obesity control group and obesity diabetes control group, the significance of the results is denoted very significant (###) if *P* < 0.001.

**Fig. 2:**
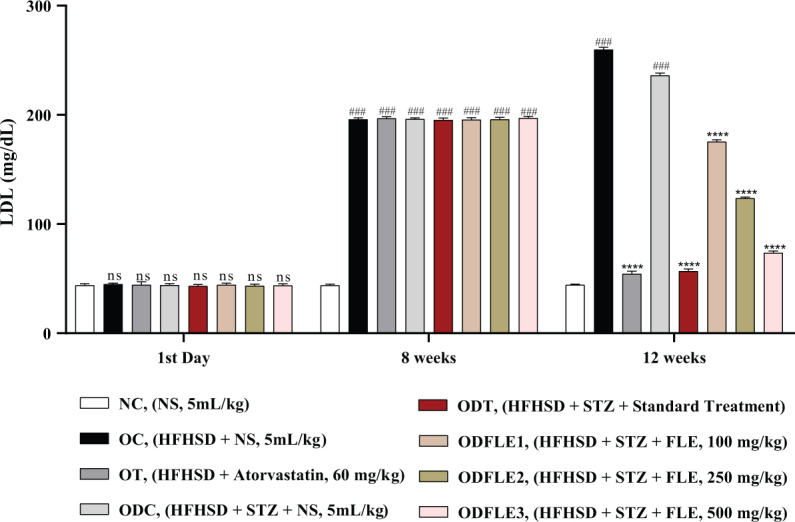
Changes in total cholesterol (TC) levels of *Wistar albino* rats STZ was administered via the intraperitoneal route after obesity induction in rats by feeding with HFHSD for 8 weeks followed by atorvastatin 60 mg/Kg bwt, glibenclamide 10 mg/Kg bwt and FLE doses at 100 mg/Kg bwt, 250 mg/Kg bwt and 500 mg/Kg bwt along with HFHSD for 4 weeks’ treatment period. (a) TC was measured through the commercial kit. Mean ± SEM of *n* = 6. Each group is analyzed using two-way ANOVA followed by Tukey’s test. At 1st day all the groups showed non-significant (ns) variations as compared to normal control (NC) group. After 8 weeks’ period, all the groups showed very significant (###) with *P* < 0.001 as compared to normal control (NC) group. After 12 weeks’ period, when the obesity control (OC) group was compared with obesity treatment (OT) group and obesity diabetes control (ODC) group is compared to treatment groups (ODT, ODFLE1, ODFLE2 and ODFLE3), results are considered non-significant (ns) if *P* > 0.05, significant (*) if *P* < 0.05, more significant (**) if *P* < 0.01, very significant (***) if *P* < 0.001, and highly significant (****) if *P* < 0.0001. When the normal control group is compared with obesity control group and obesity diabetes control group, the significance of the results is denoted very significant (###) if *P* < 0.001.

**Fig. 3:**
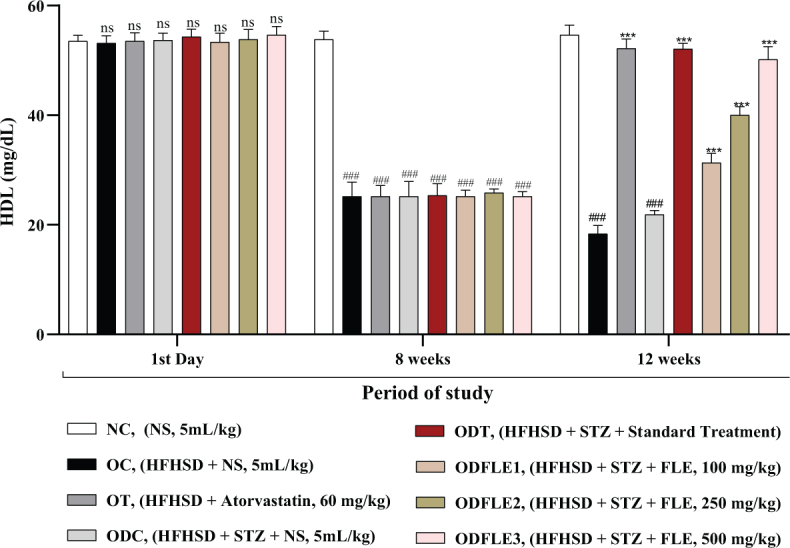
Changes in triglycerides (TG) levels of *Wistar albino* rats STZ was administered via the intraperitoneal route after obesity induction in rats by feeding with HFHSD for 8 weeks followed by atorvastatin 60 mg/Kg bwt, glibenclamide 10 mg/Kg bwt and FLE doses at 100 mg/Kg bwt, 250 mg/Kg bwt and 500 mg/Kg bwt along with HFHSD for 4 weeks’ treatment period. (a) Triglycerides level was measured through the commercial kit. Mean ± SEM of *n* = 6. Each group is analyzed using two-way ANOVA followed by Tukey’s test. At 1st day all the groups showed non-significant (ns) variations (*P* < 0.05) as compared to normal control (NC) group. After 8 weeks’ period, all the groups showed very significant (###) variations (*P* < 0.001) as compared to normal control (NC) group. After 12 weeks’ period, when the obesity control (OC) group was compared with obesity treatment (OT) group and obesity diabetes control (ODC) group is compared to treatment groups (ODT, ODFLE1, ODFLE2 and ODFLE3), results are considered non-significant (ns) if *P* > 0.05, significant (*) if *P* < 0.05, more significant (**) if *P* < 0.01, very significant (***) if *P* < 0.001, and highly significant (****) if *P* < 0.0001. When the normal control group is compared with obesity control group and obesity diabetes control group, the significance of the results is denoted very significant (###) if *P* < 0.001.

**Fig. 4:**
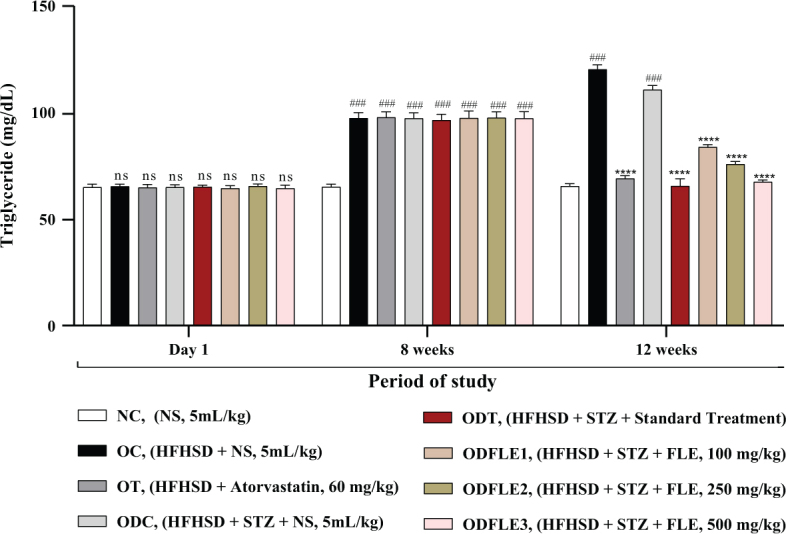
Changes in High-density lipoproteins (HDL) levels of *Wistar albino* rats STZ was administered via the intraperitoneal route after obesity induction in rats by feeding with HFHSD for 8 weeks followed by atorvastatin 60 mg/Kg bwt, glibenclamide 10 mg/Kg bwt and FLE doses at 100 mg/Kg bwt, 250 mg/Kg bwt and 500 mg/Kg bwt along with HFHSD for 4 weeks’ treatment period. (a) High-density lipoproteins were measured through the commercial kit. Mean ± SEM of *n* = 6. Each group is analyzed using two-way ANOVA followed by Tukey’s test. At 1st day all the groups showed non-significant (ns) variations (*P* < 0.05) as compared to normal control (NC) group. After 8 weeks’ period, all the groups showed very significant (###) variations (*P* < 0.001) as compared to normal control (NC) group. After 12 weeks’ period, when the obesity control (OC) group was compared with obesity treatment (OT) group and obesity diabetes control (ODC) group is compared to treatment groups (ODT, ODFLE1, ODFLE2 and ODFLE3), results are considered non-significant (ns) if *P* > 0.05, significant (*) if *P* < 0.05, more significant (**) if *P* < 0.01, very significant (***) if *P* < 0.001, and highly significant (****) if *P* < 0.0001. When the normal control group is compared with obesity control group and obesity diabetes control group, the significance of the results is denoted very significant (###) if *P* < 0.001.

**Fig. 5:**
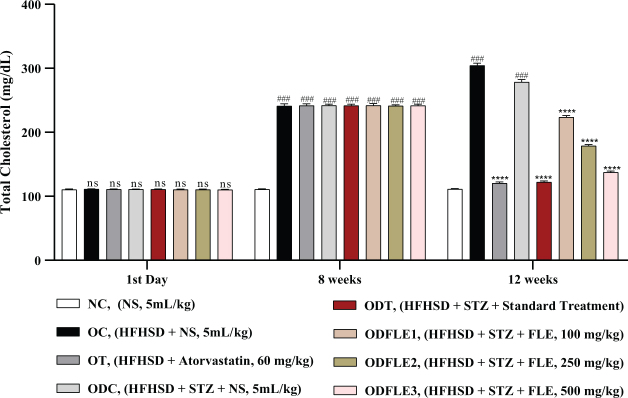
Changes in Low-density lipoproteins (LDL) levels of *Wistar albino* rats STZ was administered via the intraperitoneal route after obesity induction in rats by feeding with HFHSD for 8 weeks followed by atorvastatin 60 mg/Kg bwt, glibenclamide 10 mg/Kg bwt and FLE doses at 100 mg/Kg bwt, 250 mg/Kg bwt and 500 mg/Kg bwt along with HFHSD for 4 weeks’ treatment period. (a) Low-density lipoproteins were measured through the commercial kit. Mean ± SEM of *n* = 6. Each group is analyzed using two-way ANOVA followed by Tukey’s test. At 1st day all the groups showed non-significant (ns) variations (*P* < 0.05) as compared to normal control (NC) group. After 8 weeks’ period, all the groups showed very significant (###) variations (*P* < 0.001) as compared to normal control (NC) group. After 12 weeks’ period, when the obesity control (OC) group was compared with obesity treatment (OT) group and obesity diabetes control (ODC) group is compared to treatment groups (ODT, ODFLE1, ODFLE2 and ODFLE3), results are considered non-significant (ns) if *P* > 0.05, significant (*) if *P* < 0.05, more significant (**) if *P* < 0.01, very significant (***) if *P* < 0.001, and highly significant (****) if *P* < 0.0001. When the normal control group is compared with obesity control group and obesity diabetes control group, the significance of the results is denoted very significant (###) if *P* < 0.001.

**Fig. 6:**
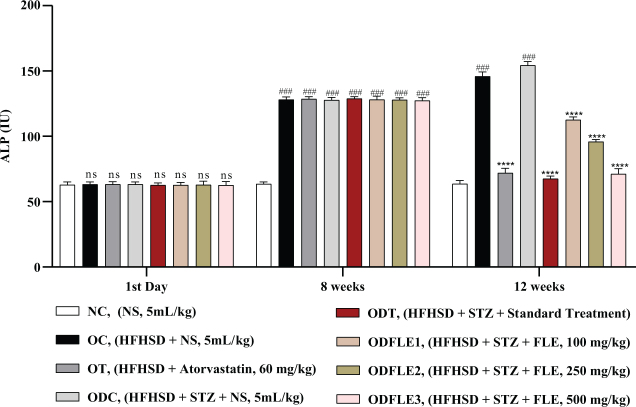
Changes in Aspartate amino transferase (AST) levels of *Wistar albino* rats STZ was administered via the intraperitoneal route after obesity induction in rats by feeding with HFHSD for 8 weeks followed by atorvastatin 60 mg/Kg bwt, glibenclamide 10 mg/Kg bwt and FLE doses at 100 mg/Kg bwt, 250 mg/Kg bwt and 500 mg/Kg bwt along with HFHSD for 4 weeks’ treatment period. (a) AST was measured through the commercial kit. Mean ± SEM of *n* = 6. Each group is analyzed using two-way ANOVA followed by Tukey’s test. At 1st day all the groups showed non-significant (ns) variations (*P* < 0.05) as compared to normal control (NC) group. After 8 weeks’ period, all the groups showed very significant (###) variations (*P* < 0.001) as compared to normal control (NC) group. After 12 weeks’ period, when the obesity control (OC) group was compared with obesity treatment (OT) group and obesity diabetes control (ODC) group is compared to treatment groups (ODT, ODFLE1, ODFLE2 and ODFLE3), results are considered non-significant (ns) if *P* > 0.05, significant (*) if *P* < 0.05, more significant (**) if *P* < 0.01, very significant (***) if *P* < 0.001, and highly significant (****) if *P* < 0.0001. When the normal control group is compared with obesity control group and obesity diabetes control group, the significance of the results is denoted very significant (###) if *P* < 0.001.

**Fig. 7:**
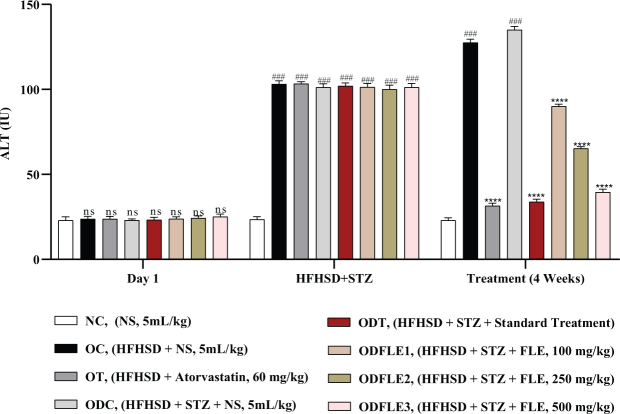
Changes in Alanine amino transferase (ALT) levels of *Wistar albino* rats STZ was administered via the intraperitoneal route after obesity induction in rats by feeding with HFHSD for 8 weeks followed by atorvastatin 60 mg/Kg bwt, glibenclamide 10 mg/Kg bwt and FLE doses at 100 mg/Kg bwt, 250 mg/Kg bwt and 500 mg/Kg bwt along with HFHSD for 4 weeks’ treatment period. (a) Alanine amino transferase was measured through the commercial kit. Mean ± SEM of *n* = 6. Each group is analyzed using two-way ANOVA followed by Tukey’s test. At 1st day all the groups showed non-significant (ns) variations (*P* < 0.05) as compared to normal control (NC) group. After 8 weeks’ period, all the groups showed very significant (###) variations (*P* < 0.001) as compared to normal control (NC) group. After 12 weeks’ period, when the obesity control (OC) group was compared with obesity treatment (OT) group and obesity diabetes control (ODC) group is compared to treatment groups (ODT, ODFLE1, ODFLE2 and ODFLE3), results are considered non-significant (ns) if *P* > 0.05, significant (*) if *P* < 0.05, more significant (**) if *P* < 0.01, very significant (***) if *P* < 0.001, and highly significant (****) if *P* < 0.0001. When the normal control group is compared with obesity control group and obesity diabetes control group, the significance of the results is denoted very significant (###) if *P* < 0.001.

**Fig. 8:**
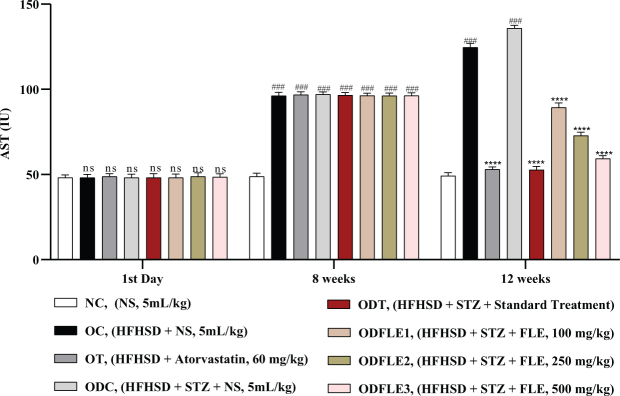
Changes in Alkaline Phosphatase (ALP) levels of *Wistar albino* rats STZ was administered via the intraperitoneal route after obesity induction in rats by feeding with HFHSD for 8 weeks followed by atorvastatin 60 mg/Kg bwt, glibenclamide 10 mg/Kg bwt and FLE doses at 100 mg/Kg bwt, 250 mg/Kg bwt and 500 mg/Kg bwt along with HFHSD for 4 weeks’ treatment period. (a) ALP was measured through the commercial kit. Mean ± SEM of *n* = 6. Each group is analyzed using two-way ANOVA followed by Tukey’s test. At 1st day all the groups showed non-significant (ns) variations (*P* < 0.05) as compared to normal control (NC) group. After 8 weeks’ period, all the groups showed very significant (###) variations (*P* < 0.001) as compared to normal control (NC) group. After 12 weeks’ period, when the obesity control (OC) group was compared with obesity treatment (OT) group and obesity diabetes control (ODC) group is compared to treatment groups (ODT, ODFLE1, ODFLE2 and ODFLE3), results are considered non-significant (ns) if *P* > 0.05, significant (*) if *P* < 0.05, more significant (**) if *P* < 0.01, very significant (***) if *P* < 0.001, and highly significant (****) if *P* < 0.0001. When the normal control group is compared with obesity control group and obesity diabetes control group, the significance of the results is denoted very significant (###) if *P* < 0.001.

**Fig. 9:**
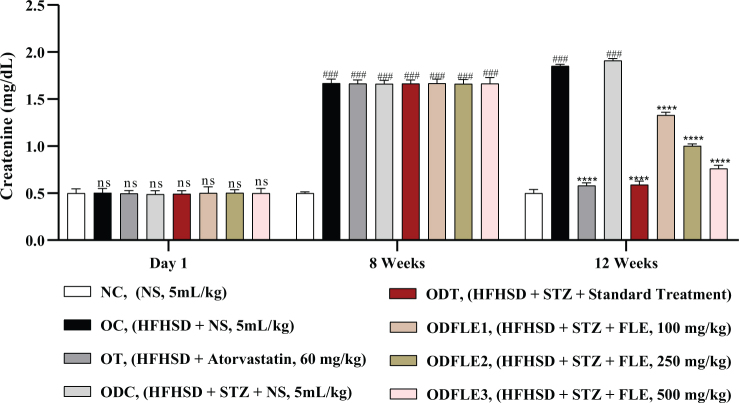
Changes in creatinine levels of *Wistar albino* rats STZ was administered via the intraperitoneal route after obesity induction in rats by feeding with HFHSD for 8 weeks followed by atorvastatin 60 mg/Kg bwt, glibenclamide 10 mg/Kg bwt and FLE doses at 100 mg/Kg bwt, 250 mg/Kg bwt and 500 mg/Kg bwt along with HFHSD for 4 weeks’ treatment period. (a) Creatinine was measured through the commercial kit. Mean ± SEM of *n* = 6. Each group is analyzed using two-way ANOVA followed by Tukey’s test. At 1st day all the groups showed non-significant (ns) variations (*P* < 0.05) as compared to normal control (NC) group. After 8 weeks’ period, all the groups showed very significant (###) variations (*P* < 0.001) as compared to normal control (NC) group. After 12 weeks’ period, when the obesity control (OC) group was compared with obesity treatment (OT) group and obesity diabetes control (ODC) group is compared to treatment groups (ODT, ODFLE1, ODFLE2 and ODFLE3), results are considered non-significant (ns) if *P* > 0.05, significant (*) if *P* < 0.05, more significant (**) if *P* < 0.01, very significant (***) if *P* < 0.001, and highly significant (****) if *P* < 0.0001. When the normal control group is compared with obesity control group and obesity diabetes control group, the significance of the results is denoted very significant (###) if *P* < 0.001.

**Fig. 10:**
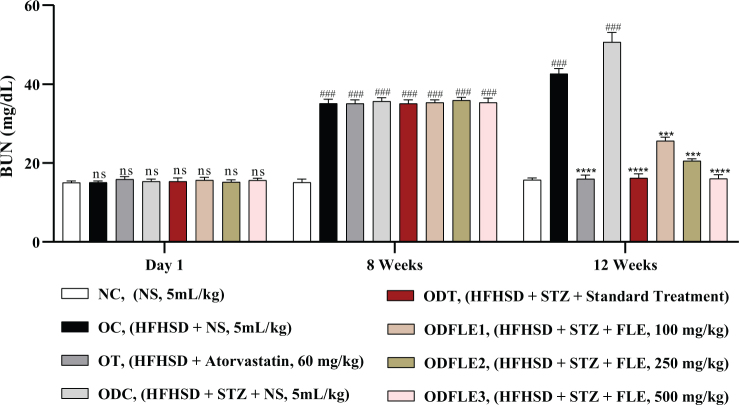
Changes in blood urea nitrogen (BUN) levels of *Wistar albino* rats STZ was administered via the intraperitoneal route after obesity induction in rats by feeding with HFHSD for 8 weeks followed by atorvastatin 60 mg/Kg bwt, glibenclamide 10 mg/Kg bwt and FLE doses at 100 mg/Kg bwt, 250 mg/Kg bwt and 500 mg/Kg bwt along with HFHSD for 4 weeks’ treatment period. (a) blood urea nitrogen was measured through the commercial kit. Mean ± SEM of *n* = 6. Each group is analyzed using two-way ANOVA followed by Tukey’s test. At 1st day all the groups showed non-significant (ns) variations (*P* < 0.05) as compared to normal control (NC) group. After 8 weeks’ period, all the groups showed very significant (###) variations (*P* < 0.001) as compared to normal control (NC) group. After 12 weeks’ period, when the obesity control (OC) group was compared with obesity treatment (OT) group and obesity diabetes control (ODC) group is compared to treatment groups (ODT, ODFLE1, ODFLE2 and ODFLE3), results are considered non-significant (ns) if *P* > 0.05, significant (*) if *P* < 0.05, more significant (**) if *P* < 0.01, very significant (***) if *P* < 0.001, and highly significant (****) if *P* < 0.0001. When the normal control group is compared with obesity control group and obesity diabetes control group, the significance of the results is denoted very significant (###) if *P* < 0.001.

**Fig. 11:**
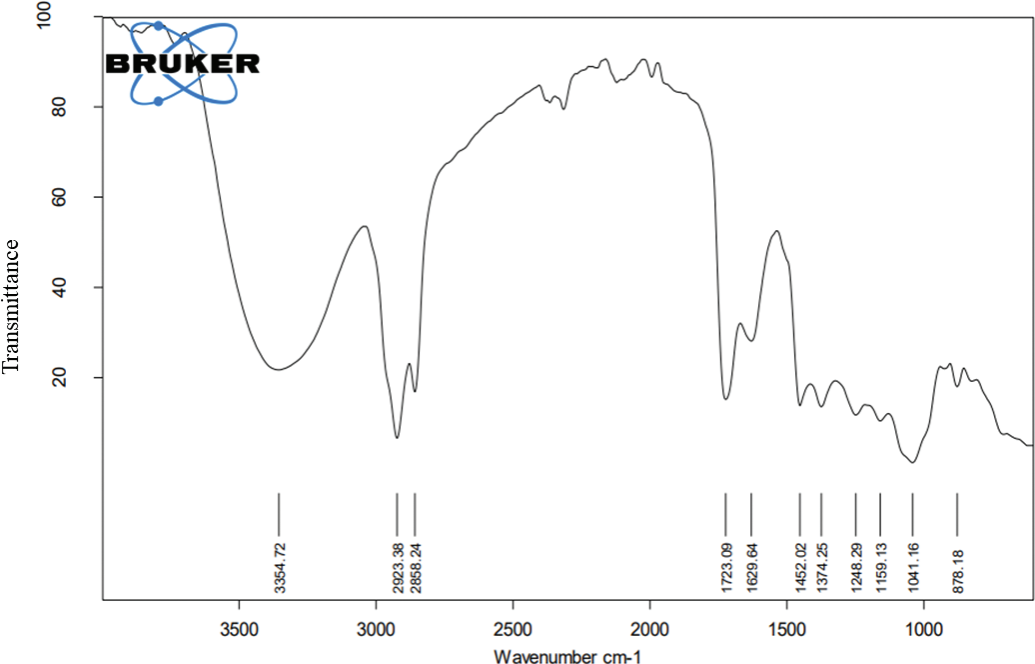
FTIR Spectra of *Ficus carica* L. ethanolic leaf extract.

**Fig. 12:**
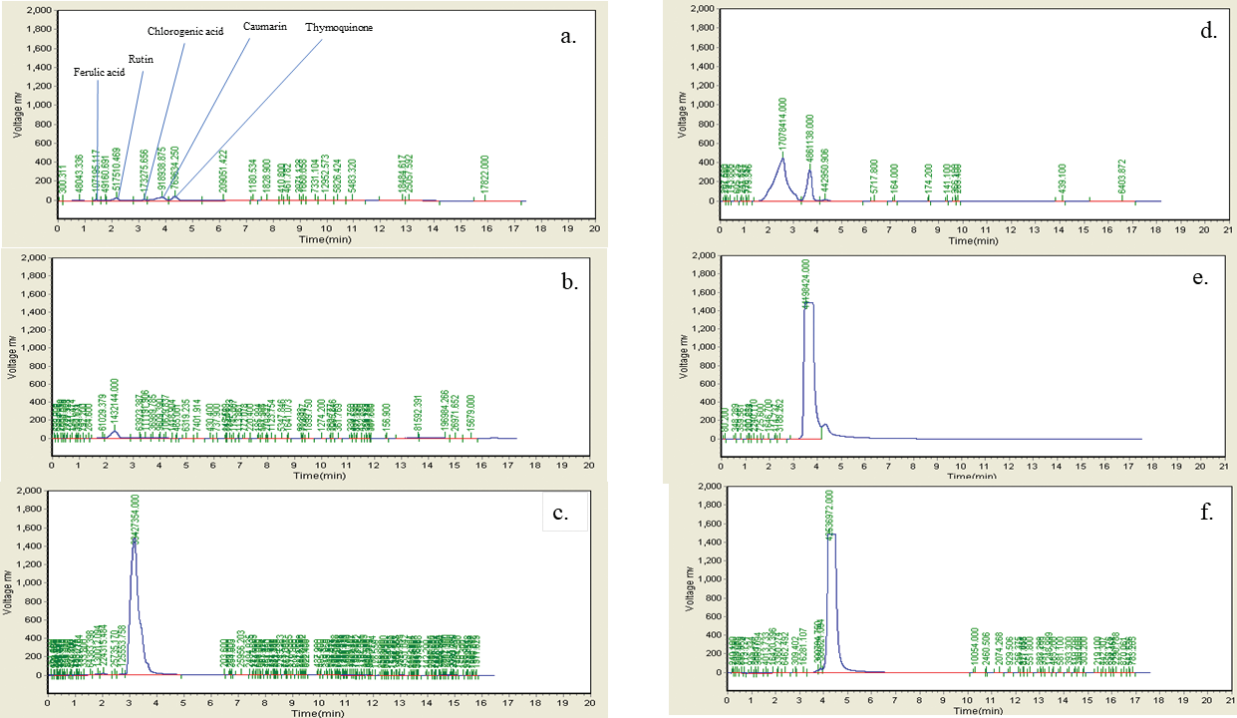
HPLC fingerprint chromatogram of *Ficus carica* L. leaf extract (a) FLE, (b) Ferulic acid, (c) Rutin, (d) Chlorogenic acid, (e) Coumarin and (f) Thymoquinone.

**Fig. 13:**
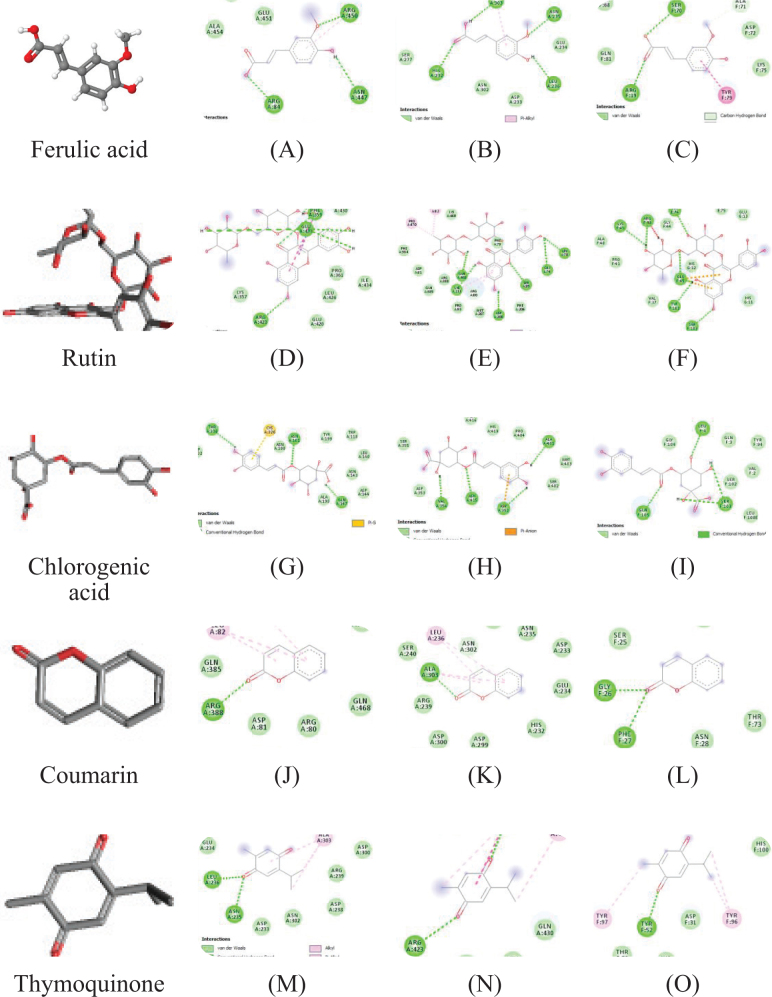
2D analysis of isolated compounds with different genes; (a) Ferulic acid with AKT1 (b) Ferulic acid with FTO (c) Ferulic acid with VEGFA (d) Rutin with AKT1 (e) Rutin with FTO (f) Rutin with VEGFA (g) Chlorogenic acid with AKT1 (h) Chlorogenic acid with FTO (i) Chlorogenic acid with VEGFA (j) Coumarin with AKT1 (k) Coumarin with FTO (l) Coumarin with VEGFA (m) Thymoquinone with AKT1 (n) Thymoquinone with FTO (o) Thymoquinone with VEGFA.

The physicochemical parameters of *Ficus carica L*. leaf powder, including the total ash value, water-soluble ash, acid-insoluble ash, and loss on drying, are presented in Table 1. Preliminary phytochemical results of the ethanolic extract are provided in Table 2, while total phenolic, total flavonoid, hydrogen peroxide, DPPH, and ferric-reducing power results are shown in Table 3. The anti-inflammatory potential of the ethanolic extract of *Ficus carica* L. leaves, assessed by different methods such as proteinase inhibition activity, heat-induced hemolysis, and BSA denaturation, is summarized in Table 4. Similarly, the results of antiglycation activity by different methods like *β*-Amyloid formation, fructosamine assay, and free carbonyl group estimation are presented in Table 5. The effect of *Ficus carica* L. leaves extract on the body weight of rats at different days is presented in Table 6. The LC-MS/MS profile of *Ficus carica* L. leaf extract is outlined in Table 7. The effects of *Ficus carica* L. ethanolic leaf extract on blood glucose levels, LDLs, HDLs, TG, and TC are depicted in Figs. 1–5, respectively. Similarly, the effect on liver parameters such as ALP, alanine aminotransferase (ALT), and AST is illustrated in Figs. 6–8, respectively. The results of the effect of the ethanolic extract on renal parameters like creatinine levels and BUN are shown in Figs. 9 and 10. FTIR spectra and HPLC fingerprint of *Ficus carica* L. leaf extract and different standard drugs are presented in Figs. 11 and 12, respectively. Molecular docking results are presented in Fig. 13.

**Table 1:** Physicochemical analysis of *Ficus carica* L. leaf

Loss on drying	Total ash value	Acid-insoluble ash	Water-soluble ash
6.20 (%)	22.48 (%)	7.68 (%)	13.57 (%)

**Table 2:** Phytochemical screening of *Ficus carica* L. ethanolic leaf extract

Sr. No.	Phytochemical constituents	Result
1	Glycosides	+
2	Terpenoids	+++
3	Tannins	+
4	Phenols	+
5	Alkaloids	+++
6	Resins	+
7	Flavonoids	++
8	Saponins	−
9	Sterol	+
10	Steroids	++
11	Proteins	++
12	Fixed oils	+
13	Gums	+
14	Mucilage	+
15	Carbohydrates	++

The symbol (–) indicates absence, (+) indicates mild concentration, (++) indicates moderate concentration, and (+++) indicates high concentration of phytoconstituent in FLE.

**Table 3:** Antioxidant analysis of ethanolic extract of *Ficus carica* L. leaves (FLE)

Sr. No.	Parameter	Plant extract	Standard
1	TPC	153 ± 2.51 mg of GAE/g	186 ± 1.23 mg of GAE/g (Gallic acid)
2	TFC	73 ± 4.01 mg RE/g	99 ± 0.43 mg RE/g (Rutin)
3	H_2_O_2_ assay	35.6 ± 0.023% inhibition	71.2 ± 0.008% inhibition (Gallic acid)
4	DPPH assay	0.58 IC_50_ (mg/mL)	0.013 IC_50_ (mg/mL) (Gallic acid)
5	FRAP assay	88.76 µg/g of FeSO_4_	

**Table 4:** Anti-inflammatory potential of *Ficus carica* L. leaves extract (FLE)

Sr. No.	Parameter	Plant extract (% inhibition)	Standard (% inhibition)
1	Proteinase inhibition activity	28 ± 0.01	84 ± 0.05
2	Heat-induced hemolysis	55 ± 0.03	86 ± 0.05
3	BSA denaturation assay	51.2 ± 0.05	54.2 ± 0.05

**Table 5:** Antiglycation potential of ethanolic extract of *Ficus carica* L. leaves (FLE)

Sr. No.	Parameter	Plant extract	Standard
1	*β*-Amyloid formation	0.017 (absorbance)	0.12
2	Fructosamine assay	19.4 ± 0.06 (% inhibition)	
3	Free carbonyl group estimation	17.0 ± 0.03 (% inhibition)	

**Table 6:** Effect of ethanolic extract of *Ficus carica* L. leaves (FLE) on body weight at different days

Sr. No.	Treatment group	Weight of animals (g)			
		1st day	8 weeks	8 weeks 2 days	12 weeks
1	NC	103.7 ± 3.65	145.2 ± 2.21	145.8 ± 2.56	171.7 ± 2.74
2	OC	108.5 ± 2.37	296.7 ± 2.56	297.9 ± 2.18	387.3 ± 5.37^#^
3	OT	103.1 ± 3.71	281.4 ± 2.35	282.6 ± 2.45	195.7 ± 2.26^***^
4	ODC	101.9 ± 2.62	296.8 ± 3.40	280.3 ± 2.98	320.8 ± 2.12^#^
5	ODT	102.3 ± 3.07	302.3 ± 2.82	293.7 ± 1.92	193.4 ± 2.12^***^
6	ODFLE1	102.4 ± 3.13	291.7 ± 2.74	278.5 ± 3.14	265.7 ± 2.86^*^
7	ODFLE2	100.7 ± 2.74	300.3 ± 5.37	284.4 ± 4.76	230.8 ± 2.24^**^
8	ODFLE3	101.3 ± 5.37	296.7 ± 2.26	281.5 ± 2.06	199.6 ± 1.98^***^

(±) indicates SEM.

**Table 7:** LC-MS/MS profile of ethanolic extract of *Ficus carica* L. leaf (FLE)

Sr.	Compound	Formula	Molecular mass g/mol	[M-H]^+^ *m/z*	Retention time (min)
1.	Nalidixic acid	C_12_H_12_N_2_O_3_	232.235	233.95	1.1
2.	(E)-2-Methoxycinnamaldehyde	C_10_H_10_O_2_	162.188	162.8	1.3
3.	Dopamine	C_8_H_11_NO_2_	153.18	155.8	1.6
4.	Xanthyletin	C_14_H_12_O_3_	228.24	230.0	1.7
5.	Herniarin	C_10_H_8_O_3_	176.16	178.8	4.6
6.	Citrinin	C_13_H_14_O_5_	250.25	251.0	6.6
7.	Oxindole	C_8_H_7_NO	133.15	134.8	6.9
8.	6-Methylcoumarin	C_10_H_8_O_2_	160.17	162.8	6.9
9.	Reynosin	C_15_H_20_O_3_	248.31	251.0	7.0
10.	Tryptamine	C_10_H_12_N_2_	160.21	161.8	7.2
11.	Xanthurenic acid	C_10_H_7_NO_4_	205.17	206.9	7.8
12.	Isoshaftoside	C_26_H_28_O_14_	564.49	565.3	9.2
13.	Homoorientin	C_21_H_20_O	448.4	449.3	9.3
14.	D-Glucuronic acid	C_6_H_10_O_7_	180.15	181.7	10.9
15.	Vanillin	C_8_H_8_O_3_	152.15	154.0	11.3
16.	Nodakenetin	C_14_H_14_O_4_	246.26	247.8	11.9
17.	4-Methylumbelliferone	C_10_H_8_O_3_	176.17	177.8	12.1
18.	Gamma-tocotrienol	C_28_H_42_0_2_	410.63	411.8	12.9
19.	Oleoylserotonin	C_28_H_44_N_2_O_2_	440.66	441.8	13.7
20.	5 *α*-androsterone	C_19_H_30_O_2_	290.44	294.1	13.8
21.	Epiandrosterone	C_19_H_30_O_2_	290.44	293.2	14.1
22.	Piperyline	C_16_H_17_NO_3_	271.316	273.1	14.8
23.	Rizatriptan benzoate	C_25_H_25_N_5_O_2_	393.51	394.1	14.9
24.	Tartaric acid	C_4_H_6_O_6_	150.87	153.8	14.9
25.	Osthol	C_15_H_16_O_3_	244.28	247.0	15.5
26.	Ribose	C_5_H_10_O_5_	150.13	153.8	15.3
27.	1,4-Dihydroxyanthraquinone	C_14_H_8_O_4_	240.21	241.1	15.7
28.	Piperine	C_17_H_19_NO_3_	285.35	286.1	16.1
29.	Averufin|Averufine	C_20_H_16_O_7_	368.08	367.2	16.4
30.	(+/-)-Alpha-lipoic acid amide	C_8_H_15_NOS_2_	205.33	206.9	17.5
31.	Myosmine	C_9_H_10_N_2_	146.19	147.8	18.0
32.	Avenanthramide D	C_16_H_13_NO_4_	283.28	285.1	18.5
33.	Stearidonic acid	C_18_H_28_O_2_	276.4	277.0	18.5
34.	Flunixine	C_14_H_11_F_3_N_2_O_2_	296.24	297.2	20.0
35.	Indoxyl sulfate	C_8_H_7_NO_4_S	213.21	214.8	22.3
36.	Indole-3-acetaldehyde	C_10_H_9_NO	158.18	158.8	22.5
37.	Linolenic acid ethyl ester	C_20_H_36_O	308.49	309.7	22.7
38.	Pheophorbide A	C_35_H_36_N_4_O	592.68	593.4	24.3
39.	Baccatin III	C_31_H_38_O_11_	586.62	587.4	26.0
40.	Genipin 1-gentiobioside	C_23_H_34_O_15_	550.5	551.4	27.3

Ferulic acid, rutin, chlorogenic acid, coumarin, and thymoquinone are the five significant active ingredients that were selected for molecular docking verification with AKT1, VEGFA, and FTO as the primary targets, as shown in Fig. 13. These targets are thought to be promising for the treatment of hyperlipidemia, diabetes, inflammation, and obesity.

In order to quantify the bonding activity between the docking molecules, AutoDock Vina computed the binding energy, or Vina score, as shown in Table 8. A smaller Vina score denotes a stronger bonding activity, better affinity, and more stable structure between the ligand and the receptor.

**Table 8:** The binding energies (kcal/mol) of docked ligand–protein complex calculated with AKT1, FTO, and VEGFA

Compounds	Genes	Pose No.	Docking score (kcal/mol)	Hydrogen bonds	Electrostatic/hydrophobic bonds
Ferulic acid	AKT1	8	−4.0	Arg450, Asn447, Arg84	Ala454, Arg455, Glu451
Rutin		4	−6.9	Ser358, Phe359, Glu433, Arg423	Glu360, Gln430, Pro361, Ile434, Leu426, Glu420, Lys357
Chlorogenic acid		3	−5.1	Thr330, Asn101, Gln147	Asp332, Cys326, Asn200, Tyr199, Trp118, Leu140, Asn143, Asp144, Ala198
Coumarin		1	−5.8	Arg388	Gln385, Leu82, Pro470, Phe384, Lys469, Gln468, Arg80, Phe79, Asp81
Thymoquinone		6	−3.9	Leu236, Asn235	Glu234, Ala303, Asp300, Arg239, Asp238, Asn302, Asp233
Ferulic acid	FTO	5	−4.6	Ala303, Asn235, Leu236, His232	Glu234, Asp233, Asn302, Ser277
Rutin		1	−8.1	Leu78, Gly76, Ser95, Asp208, Lys211, Gln468	Phe384, Pro470, Leu82, Lys469, Leu464, Leu91, Thr463, Lys391, Phe79, Phe206, Met207, Arg80, Pro93, Gln385, Arg388, Asp81
Chlorogenic acid		4	−4.8	Ala481, Asp352, Asn415, Val354	Ser355, Ala416, His419, Pro484, Met483, Ser482, Asp353
Coumarin		1	−5.5	Ala303	Ser240, Leu236, Asp238, Asn302, Val237, Asn235, Asp233, Glu234, His232, Asp299, Asp300, Arg239
Thymoquinone		2	−4.1	Phe359, Arg423	Ser358, Glu360, Ile434, Gln430, Leu426, Glu420
Ferulic acid	VEGFA	2	−4.9	Ser70, Arg19	Thr68, Ala71, Asp72, Lys74, Tyr79, Gln81
Rutin		1	−7.4	Lys43, Arg39, Ser74, Glu45, Tyr101, Ser103	Ala40, Gly44, Asn100, Gln98, Asn74, Ile76, Glu13, His12, His11, Val37, Pro41
Chlorogenic acid		5	−4.3	Leu4, Ser103, Gln105	Gly104, Gln3, Tyr94, Val2, Ser102, Leu100E
Coumarin		2	−3.9	Gly26, Phe27	Ser25, Asn76, Thr73, Asn28
Thymoquinone		1	−4.6	Tyr12	Trp99, His100, Tyr96, Asp31, Lys30, Thr53, Tyr97

## Discussion

Physicochemical evaluation of crude drug materials is considered as the very important parameter for the development of standardized quality control profile of herbal medicine according to World Health Organization. Physicochemical standards play a crucial role in assessing the authenticity, purity, quality, and efficacy of drug materials. Proper control or minimization of moisture content is essential to prevent the decomposition of crude drugs, whether it is due to microbial contamination or chemical changes. Ideally, maintaining a moisture content range of 10–20% is considered optimal for reducing fungal and bacterial growth (26). In the current research work, crude leaf powder of *Ficus carica* L. contained 6.2017% of moister content. The identification and purity of crude drugs can also be determined by the ash value analysis. In this study, the total ash value was found to be 22.48%, the water-soluble ash value is 13.57%, and the acid-insoluble ash value was 7.68%. Secondary metabolites obtained from plants are considered important as a source for developing innovative therapeutic agents, and researchers worldwide are increasingly turning to medicinal plants to exploring new bioactive compounds (27). In this study, the phytochemical analysis of the ethanolic extract of *Ficus carica* L. leaves revealed the presence of several important metabolites, including tannins, phenols, glycosides, flavonoids, resins, alkaloids, steroids, and terpenoids. These compounds are biologically active and contribute to the pharmacological and physiological activities of the plant. Phenolic compounds, derived from tyrosine and phenylalanine, are considered significant secondary metabolites due to their scavenging ability. In our research, the phenolic content in the leaves of *Ficus carica* was found to be 153 ± 2.51. Among these phenolic compounds, flavonoids represent the largest and most important class. Flavonoids are known for their valuable effects on human health, including anticancer, antibacterial, anti-inflammatory, anti-allergic, and antiviral activities. They are also highly effective scavengers of oxidizing molecules such as singlet oxygen. In our study, the total flavonoid content in the *Ficus carica* leaf was found to be 73 ± 4.01 (mg RE/g). Assessing the antioxidant capacity of an extract requires the use of multiple antioxidant assays to understand its various mechanisms of action. Therefore, we employed different antioxidant activity assays, including DPPH, ferric-reducing antioxidant power, and hydrogen peroxide assays, to evaluate the antioxidant potential of *Ficus carica* L. leaf extract. The DPPH radical scavenging assay is widely used to assess the free radical scavenging potential of extracts or compounds. Compounds with lower EC_50_ values are considered to have higher antioxidant potential. In our research, the EC_50_ value of *Ficus carica* L. leaf extract was determined to be 0.58 mg/mL. This could be attributed to the extract’s ability to donate hydrogen atoms, thereby forming stable compounds from free radicals and inhibiting the oxidation process. Hydroxyl radicals (OH) generated from the decomposition of hydrogen peroxide naturally occur in microorganisms, food, plants, and the human body. They initiate lipid peroxidation and cause DNA damage. The ethanolic extract of *Ficus carica* leaf effectively scavenged hydrogen peroxide due to its phenolic groups, which neutralize it into water by donating electrons (28).

Several diseases are linked with oxidative stress, which becomes elevated due to increased rates of metabolic pathways (29). Antioxidants play a pivotal role in mitigating oxidative stress, making them highly beneficial in treating various health conditions. The FRAP assay is a valuable method for assessing oxidative stress as it evaluates the ferrous reducing antioxidant power of a given sample. This assay measures the ability of antioxidants to scavenge free radicals and reduce the Fe (III)/tripyridyltriazine complex.

In our study, the FRAP value of the *F. carica* ethanolic extract was moderate, measuring 88.76 µg/g of FeSO4. This indicates that the extract possesses the capacity to reduce oxidative stress. However, a relatively low FRAP value suggests that the sample contains lower levels of phenolic compounds (30). Both obesity and diabetes are conditions associated with oxidative stress. Therefore, the use of antioxidants may reduce diabetes and obesity-induced complications.

We investigate *F. carica* extract for its protective effects against denaturation of BSA, and a significant activity (51.2 ± 0.05%) was recorded, which indicated a strong protective capacity of tested sample. Numerous studies have confirmed that tissue damage in the body is primarily facilitated by proteinases during inflammatory processes. Therefore, the inhibition of proteinases can be beneficial in the management of inflammatory responses (31). In our case, lower levels of inhibition (28 ± 0.01%) was recorded, which is an indication of little contribution in this mechanism. Similarly, inflammatory modulation is also attributed to the release of lysosomal contents resulting from cellular breakage (32). Given the close resemblance of the cell membrane of red blood cells to lysosomes (33), we assessed the protective effect of the tested samples on heat-induced hemolysis. The results revealed a moderate protective effect (55 ± 0.03%) of the tested sample on red blood cells.

Advanced glycation end products (AGEs) play a crucial role in the development of diabetic complications, including both microvascular and macrovascular complications. There are several types of AGEs that play key role in progression of diabetic pathologies, including *β*-Amyloid Formation, Fructosamine adduct formation, and free carbonyl formation (34). We analyzed the *F. carica* extract, and it was evident that tested sample has lower levels of absorbance compared to control (BSA + glucose). It was considered as having protective role on AGEs formation. However, in case of Fructosamine assay and free carbonyl estimation assay, lower inhibition levels were recorded. It was, therefore, concluded that *F. carica* may have moderate levels of AGEs inhibition.

HFHSD/streptozotocin (STZ)-induced rodent diabetic models have gained popularity due to their ability to mimic both obesity and diabetes, which can be adjusted by varying the duration of HFHSD administration (35). In our study, we effectively established an obesity diabetes model in rats by combining an 8-week HFHSD with a single dose of STZ, albeit with minor modifications. The obesity diabetes model exhibited a notable increase in fasting blood glucose levels compared to normal rats (36). Throughout the study, body weights were consistently monitored, revealing a significant increase in the ODC group, signifying the development of obesity in the obesity diabetes model. This increase is attributed to metabolic abnormalities and an augmentation in adipose tissue mass, characterized by hyperglycemia, hyperinsulinemia, and hyperleptinemia (37). Interestingly, despite the significant increase in body weights observed in the ODC group, the actual weight gain in grams was lower compared to the OC group, which received the high-fat high-sucrose diet alone for a similar duration (38). *Wistar albino* rats showed significant increase (*P* < 0.005) in body weights of OC and ODC groups. After treatment with FLE (100, 250, and 500 mg/Kg), there was substantial (*P* < 0.001) reduction in body weight in treatment groups of OT, ODT, ODFLE1, ODFLE2, and ODFLE3. The variation in body weight of animals during the study period is summed up in Table 6. This discrepancy may be attributed to the effect of STZ, which has been shown in previous studies to limit weight gain (39). FLE reduced the body weight, and this reduction was more pronounced in the ODFLE3 group comparing to lower doses of FLE.

Blood glucose level showed a significant increase (*P* < 0.001) in ODC groups. After treatment with FLE at the dose of 100, 250, and 500 mg/Kg of body weight substantially (*P* < 0.001), it lowers the blood glucose level depending upon the dose in ODFLE1, ODFLE2, and ODFLE3 groups when compared with the ODC group. FLE significantly reduced blood glucose levels at higher doses of 500 mg/Kg shown in Fig. 1.

Serum TC level showed a substantial increase (*P* < 0.001) in obesity control (OC) and ODC groups. After treatment with FLE (100, 250, and 500 mg/Kg), a momentous (*P* < 0.001) reduction in TC in ODFLE1, ODFLE2, and ODFLE3 groups was noted depending upon the dose in comparison to the ODC group in Fig. 2.

Substantial increase (*P* < 0.001) in the TG level was observed in the OC and ODC groups. After treatment with FLE (100, 250, and 500 mg/Kg of body weight), there was momentous (*P* < 0.001) lessening of the TG level in ODFLE1, ODFLE2, and ODFLE3 when compared with the ODC group, which is shown in Fig. 3. Serum HDL showed decrease (*P* < 0.001) in the OC and ODC groups. After treatment with FLE (100, 250, and 500 mg/Kg body weight), a significant (*P* < 0.001) increase in the HDL level was observed in the ODFLE1, ODFLE2, and ODFLE3 groups in a dose-dependent manner when compared with the ODC group, as shown in Fig. 4.

There was an increase (*P* < 0.001) in the serum LDL level in the OC and ODC groups. After treatment with FLE (100, 250, and 500 mg/Kg of body weight), there was a substantial decrease (*P* < 0.001) in the LDL level in the ODFLE1, ODFLE2, and ODFLE3 groups, respectively, depending upon the dose in comparison to the ODC group, as shown in Fig. 5.

The level of serum AST increased (*P* < 0.001) significantly in the OC and ODC groups. After treatment with FLE (100, 250, and 500 mg/Kg), a significant decline (*P* < 0.001) was observed depending on the dose in the AST level in different groups, such as ODFLE1, ODFLE2, and ODFLE3, when compared with the ODC group, as shown in Fig. 6.

The serum ALT level was elevated (*P* < 0.005) in the OC and ODC groups. After treatment with different doses of FLE, there was a significant (*P* < 0.005) decrease in the ALT level in the dose-dependent manner in the ODFLE1, ODFLE2, and ODFLE3 groups when compared with the ODC group (Fig. 7). Serum ALP level showed a significant increase (*P* < 0.005) in the OC and ODC groups. After treatment with FLE (100, 250, and 500 mg/kg), a substantial decline (*P* < 0.005) in the ALP level was estimated in the ODFLE1, ODFLE2, and ODFLE3 groups depending upon the dose when compared with the ODC group, as shown in Fig. 8.

The Serum Creatinine level showed a significant increase (*P* < 0.005) in the OC and ODC groups. After treatment with FLE (100, 250, and 500 mg/Kg), a noteworthy decline (*P* < 0.005) in the creatinine level was estimated in the ODFLE1, ODFLE2, and ODFLE3 groups depending upon the dose, as shown in Fig. 9. BUN level showed a significant increase (*P* < 0.005) in the OC and ODC groups. After treatment with FLE (100, 250, and 500 mg/kg), a substantial decline in the BUN level (*P* < 0.005) was observed in a dose-dependent manner in different groups (ODFLE1, ODFLE2, and ODFLE3) in comparison to the ODC group, as shown in Fig. 10.

On FTIR, the difference between the spectra considered as a proof of transformation. The band observed in 3,354 cm^-1^ as alcohol (O—H stretching) or as aliphatic primary amines (N—H stretching), 2,923 and 2,858 cm^-1^ as alkanes (C—H Stretching), s/cm as aldehyde (—CHO) 1740-1725, 1,723 cm^-1^ as aldehyde (—C=O Stretching), 1,629 cm^-1^ as alkene (C=C), 1,452 cm^-1^ as alkane (C—H), 1,374 cm^-1^ as phenol or alcohol (Ph—OH/ O-H Bending), 1,248 cm^-1^ as amine (C—N Stretching), 1,159 cm^-1^ as tertiary alcohol (C—O), 1,041 s/cm as anhydride (CO—O—CO) or sulfoxide (S=O), and 878 s/cm as trisubstituted or disubstituted alkanes (C—H bending) are labeled in the extracted spectrum. HPLC chromatogram depicts the presence of rutin, chlorogenic acid, coumarin, and thymoquinone in which the presence of coumarin was identified in Innocenti et al. (40), whereas rutin and chlorogenic acid peaks in the chromatogram of FLE are previously identified in Teixeira et al. (41). FLE also revealed rich source of alkaloids, coumarin, terpenoids, steroids, and carbohydrates, which showed in LC-MS/MS chromatographic results; all the phytochemicals exhibit marked antioxidant potentials, and previous studies enumerate the potential use of FLE for diabetics and other oxidative stress-induced diseases (42).

The standardized leaf extract of *Ficus carica L*. was subjected to profiling and analysis using LC-MS/MS in both negative and positive ionization modes to qualitatively characterize its constituents. To our best knowledge, we are using LC-MS/MS for the first time for qualitative analysis to detect compounds in FLE extract. Characterization of compounds was carried out by absorption spectrum in the UV-visible region, retention times, spectrum obtained by fragmentation profile, MS-ESI, and comparison with the aforementioned literatures (Table 7). The LC-MS/MS analysis results allowed the tentative assignment of constituents with the help of positive ionization mode. In the present research work, the positive mode ESI was more sensitive for the identification of alkaloids, flavonoids, terpenoids, and coumarins in the extract.

ADMET analysis is a significant method in drug development. The Swiss ADME database was utilized for ADMET analysis, and the results demonstrated that these 5 compounds have outstanding pharmacokinetic qualities (43). As shown in Table 9, none of the five identified drug candidates had any pharmacokinetic side effects in various prediction models, which included P-glycoprotein substrates, blood–brain barrier penetration, gastrointestinal absorption, and human oral absorption. Furthermore, all of the main active ingredients demonstrated good biocompatibility in each of the toxicity prediction models, with none exhibiting hazardous behavior. In renal and respiratory predictions, ferulic acid, rutin, and chlorogenic acid exhibit active properties. The toxicity radar graphic indicates that there is little confidence in positive toxicity outcomes (44, 45).

**Table 9:** ADMET profiling of identified compounds

Properties	Compounds				
	Ferulic acid	Rutin	Chlorogenic acid	Coumarin	Thymoquinone
TPSA [A^0^]	66.76	269.43	164.75	30.21	34.14
Consensus Log *P*_O/W_	1.36	−1.29	−0.38	1.82	1.85
Water solubility [logmol/L]	−2.11	−3.30	−1.62	−2.29	−2.18
GI absorption	High	Low	Low	High	High
Skin permeability [log Kp]	−6.41	−10.26	−8.76	−6.20	−5.74
P-glycoprotein substrate	No	Yes	No	No	No
BBB permeability	Yes	No	No	Yes	Yes
CYP2D6 substrate	No	No	No	No	No
CYP3A4 substrate	No	No	No	No	No
CYP1A2 inhibitor	No	No	No	Yes	No
CYP2C19 inhibitor	No	No	No	No	No
CYP2C9 inhibitor	No	No	No	No	No
CYP2D6 inhibitor	No	No	No	No	No
CYP3A4 inhibitor	No	No	No	No	No
Hepatotoxicity	No	No	No	No	No
Neurotoxicity	No	No	No	Yes	No
Nephrotoxicity	Yes	Yes	Yes	No	No
Respiratory toxicity	No	Yes	Yes	No	No
Cardiotoxicity	No	No	No	No	No
Carcinogenicity	No	No	No	Yes	No

In recent times, molecular docking technique has been utilized to search for active ingredients and probable targets of action, to assess the degree of compound-target interaction, and to interpret the pharmacological processes of action used by conventional medications (46). The top protein–ligand complexes in each category with the most hydrogen bonding were chosen based on molecular docking data and docking affinity. This study’s findings can be used to guide the initial screening of active compounds in *Ficus carica* L., as well as to provide a unique therapeutic approach for further investigation of the mechanisms of action for hyperlipidemia, antidiabetes, anti-inflammatory, and obesity.

## Conclusion

This research work concluded that the *F. carica* L. ethanolic leaf extract possesses anti-obesity and antidiabetic potential for the long-term treatment in rats with obesity and diabetes due to its antioxidants, phenolic, and flavonoids present in the extract. The findings regarding biochemical parameters supported the anti-obesity and antidiabetic efficacy of *Ficus carica* L. leaf extract, as evidenced by reductions in serum lipid profile and liver enzyme levels, alongside enhancements in renal function.

## Data Availability

The information used to substantiate the conclusions of this study can be obtained from the corresponding author upon request.

## Declaration

The Institutional Animal Ethics Committee, Faculty of Pharmacy and Pharmaceutical Sciences, University of Karachi, approved the experiment’s protocol, and the experiment was conducted in accordance with the committee rules under Institutional Bioethical Committee Approval No. IBC KU-289/2022.
